# Controlling the Mechanical Properties of Bulk Metallic Glasses by Superficial Dealloyed Layer

**DOI:** 10.3390/nano7110352

**Published:** 2017-10-27

**Authors:** Chaoyang Wang, Man Li, Mo Zhu, Han Wang, Chunling Qin, Weimin Zhao, Zhifeng Wang

**Affiliations:** 1 School of Materials Science and Engineering, Hebei University of Technology, Tianjin 300130, China; chaoyangwang@yahoo.com (C.W.); mlimail2017@163.com (M.L.); zhumomail@163.com (M.Z.); wanghan0603@163.com (H.W.); wmzhao@yahoo.com (W.Z.); 2 Key Laboratory for New Type of Functional Materials in Hebei Province, Hebei University of Technology, Tianjin 300130, China

**Keywords:** metallic glasses, dealloying, porous metals, mechanical properties

## Abstract

Cu_50_Zr_45_Al_5_ bulk metallic glass (BMG) presents high fracture strength. For improving its plasticity and controlling its mechanical properties, superficial dealloying of the BMG was performed. A composite structure containing an inner rod-shaped Cu-Zr-Al amorphous core with high strength and an outer dealloyed nanoporous layer with high energy absorption capacity was obtained. The microstructures and mechanical properties of the composites were studied in detail. It was found, for the first time, that the mechanical properties of Cu_50_Zr_45_Al_5_ BMG can be controlled by adjusting the width of the buffer deformation zone in the dealloyed layer, which can be easily manipulated with different dealloying times. As a result, the compressive strength, compressive strain, and energy absorption capacity of the BMGs can be effectively modulated from 0.9 to 1.5 GPa, from 2.9% to 4.7%, and from 29.1 to 40.2 MJ/m^3^, respectively. The paper may open a door for developing important engineering materials with regulable and comprehensive performances.

## 1. Introduction

Bulk metallic glasses (BMGs) represent an interesting group of materials as they possess superior strength and useful features compared to their crystalline counterparts [1,2]. However, BMGs fail by the formation of highly localized shear bands, which leads to catastrophic failure without much macroscopic plasticity [3,4]. This inhomogeneous deformation behavior has so far seriously limited the application of BMGs. To solve this problem, there have been various attempts to produce reinforced second phases BMG composites [5,6,7] or pores containing BMG foams [8,9]. More recently, a new group of thin-film metallic glasses (TFMGs) has also been reported to exhibit a ductile mechanical behavior. The brittle-like behavior and the development of the shear band are mitigated when the sample size is reduced down to the submicron scale [10,11,12,13]. By the combination of a unique porous structure and good mechanical properties as well as sound/energy absorption, porous BMGs are particularly considered to have many multifunctional applications such as in lightweight structures, energy absorbers, or biomaterials [14,15].

To date, many studies [16,17,18] have been based on Pd- and Zr-based BMGs systems, as exemplified for Pd-Cu-Ni-P, Zr-Ti-Ni-Cu-Be, and Zr-Al-Ni-Cu BMGs, to fabricate either open-pore type or close-cellular glassy alloys, because the Pd- and Zr-based glassy alloy systems exhibit very high glass-forming ability. The synthesis methods of the porous glassy alloys were often utilized by liquid state processing, e.g., infiltration of a temporary space-holder phase [14,19], and expansion of entrapped gas in the liquid phase or by powder extrusion with a fugitive second phase [20,21]. Compared with the glassy metals, these porous glassy alloys revealed much higher compressive ductility [14,22,23] with non-catastrophic damage accumulation [24]. However, the pore sizes of the porous glassy alloys with various techniques lie in the micrometer scale of 20–400 μm. There have been rare studies on producing 3D nanoporous BMGs. Moreover, the current fabrication processes are very complicated and costly, which makes it less competitive in commercial application. Therefore, it is necessary to apply an ultra-simple fabrication technology to develop a new type of 3D nanopores/BMG.

Recently, we introduced one-pot chemical dealloying technique applied on the Cu-Zr-Al BMG to facilely synthesize a NPC/BMG composite rod (NPC/BMG: nanoporous copper/bulk metallic glass) with a hierarchical nanoporous structure and enhanced compressive strain [25]. Compared to the previous multi-step strategy [19,20,21], the one-pot route has the evident advantages of simplicity and economy. The microstructure, mechanical properties, and fracture deformation of a NPC/BMG composite rod, obtained by chemical dealloying the Cu_50_Zr_45_Al_5_ BMG in HF solution for one day, is discussed in our previous work [25]. However, there still remain several unclear issues for the new-type nanoporous BMG composites. Does the thickness of dealloyed nanoporous copper (NPC) layer influence the mechanical properties and fracture morphologies? How does the interaction between the NPC superficial layer and the inner BMG matrix core change upon the dealloying time? In order to solve the puzzling problems, the present work aims to investigate the effect of dealloying time on dealloyed nanoporous structure, their mechanical properties, and their energy absorption capacity (EAC). The correlation among the thickness of NPC layer, the width of transition zone and the compressive strain is established in this work. The work present here will provide considerable insight into the NPC/BMG composites and pave the way for their application as a new advanced engineering material.

## 2. Materials and Methods

Master alloys with nominal compositions of Cu_50_Zr_45_Al_5_ (at %) were fabricated from pure Cu, Zr, and Al metals with 99.99 mass% purity by arc melting under an argon atmosphere using a water-cooled Cu hearth. The master ingots were remelted and then ejected into copper mold to produce a 40-mm-long bulk glassy rod with a diameter of 2 mm. The cylindrical specimens were machined into 4 mm in height and then polished. The dealloying technique [25,26,27] was performed in 0.05 M HF aqueous solution at room temperature (RT, ~298 K) with the duration ranging from one day to 5 days. Meanwhile, three NPC/BMG composite rods prepared with dealloying time t = 1, 3, and 5 days, is labeled as NPC/BMG rod 1, 2, and 3, respectively. After dealloying, the samples were rinsed with distilled water and dehydrated alcohol, and then kept in a vacuum chamber to avoid oxidation. The phase structure of the as-cast and as-dealloyed rods was identified by X-ray diffraction (XRD, D8-Advance, Bruker, Germany) analysis using Cu Kα radiation. The working current, voltage and, scan speed in XRD analysis is 30 mA, 40 kV, and 2°/min, respectively. Scanning electron microscopy (SEM, Hitachi S-4800, Tokyo, Japan) attached with energy dispersive X-ray spectroscopy (EDS, Bruker, Karlsruhe, Germany) was applied to examine the changes in the microstructure and morphology of the as-dealloyed specimens upon dealloying. The compression mechanical test at room temperature was performed on a universal mechanical testing machine (Instron 5500, Norwood, MA, USA) with the strain rate of 5.0 × 10^−4^ s^−1^.

## 3. Results and Discussion

### 3.1. Structural Hierarchy of NPC/BMG Composite Rods

**Figure 1:**
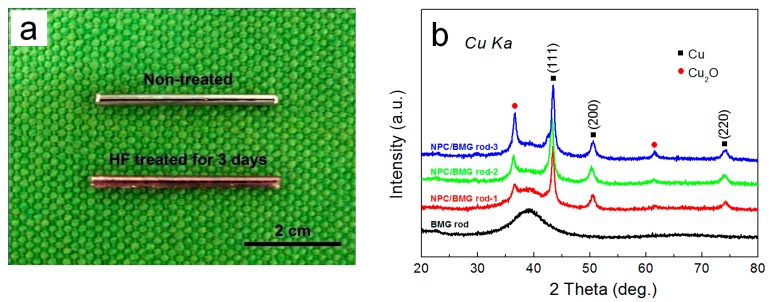
(**a**) Photos (scale bar, 2 cm) and (**b**) X-ray diffraction (XRD) patterns of precursor Cu_50_Zr_45_Al_5_ alloys rods before and after dealloying in 0.05 M HF solution for 1, 3 and 5 days at 298 K, respectively.

The photographic images and the XRD patterns of the as-cast Cu_50_Zr_45_Al_5_ rods and their as-dealloyed samples are shown in Figure 1. From Figure 1a, it is observed that the dealloyed rods after immersion for three days display perfect Cu metallic shinning luster and good mechanical integrity. Furthermore, the XRD pattern (Figure 1b) of the as-cast Cu_50_Zr_45_Al_5_ rod presents a characteristic broad halo with the absence of crystalline peak, thus indicating a glassy structure thereof. After dealloying, some sharp diffraction peaks overlapping on glassy halo peak are identified as the face-centered cubic (fcc) Cu phase and minor Cu_2_O crystalline phase. The results indicate that fcc Cu metal is formed on the Cu-Zr-Al glassy matrix by preferentially dissolving the Al and Zr constituent elements into HF solutions [28], thus exhibiting that the nanoporous copper plus a glassy matrix (NPC/BMG composite rods) is fabricated by chemical-free dealloying in 0.05 M HF solution for one to five days.

**Figure 2:**
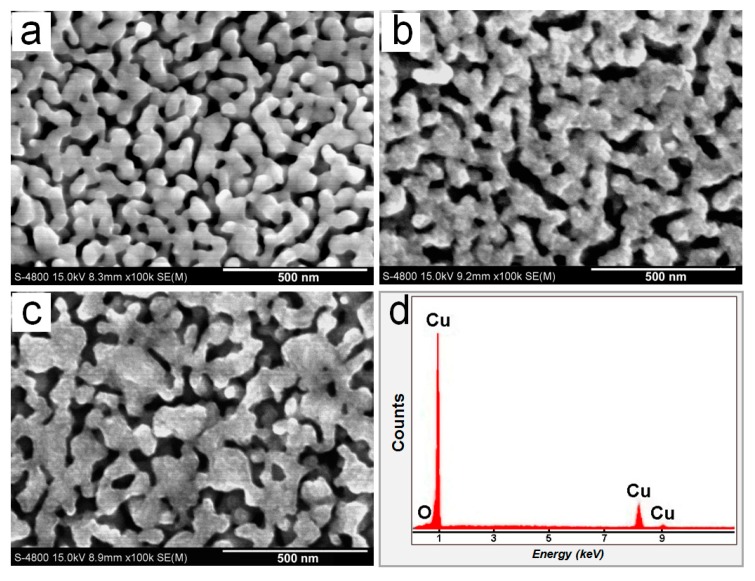
(**a**–**c**) Scanning electron microscopy (SEM) images showing the surface microstructure of the Cu_50_Zr_45_Al_5_ bulk metallic glass (BMG) immersed in the 0.05 M HF solution for 1, 3, and 5 days at 298 K, respectively; (**d**) energy dispersive X-ray spectroscopy (EDS) spectra of the nanoporous surface in (**b**).

Figure 2 shows the nanoporous structure of the surface of the NPC/BMG composite rods and a typical EDS result. SEM images (Figure 2a–c) illustrate that the homogeneous three-dimensional (3D) nanoporous morphologies with continuous pore channels and solid ligaments are formed for the Cu_50_Zr_45_Al_5_ BMG rods immersed in 0.05 M HF for 1, 3, and 5 days. A typical EDS result indicates that the elemental compositions of the porous surfaces mainly consist of Cu. After dealloying in 0.05 M HF for one day, the NPC (Figure 2a) exhibits the mean ligament width of 118 nm and pore size of 45 nm. However, the mean ligament width of the NPC formed for three and five days increases to 128 and 138 nm, respectively, indicating that the ligament becomes coarse with the prolongation of etching time. This behavior is in agreement with previous report [29]. In the HF corrosive solution, Al and Zr metals exhibit high electrochemical activity, whereas Cu metal possesses much higher stability. Therefore, the formation mechanism of the NPC dealloyed from the Cu-Zr-Al metallic glasses is mainly controlled by fast dissolution of less noble atoms (Al and Zr atoms) and the self-assembly of residual Cu atoms at alloy/electrolyte interfaces [28]. For a long leaching time in the HF solution, the overwhelming majority of Zr and Al are almost leached out at the outer part of the glassy precursor rod, whereas the Cu atoms would diffuse far enough to form more stable and coalesced nanostructure. Thus, the early formed ligaments of NPC on the surface become ripened and coarse with further immersion in the HF solution. 

**Figure 3:**
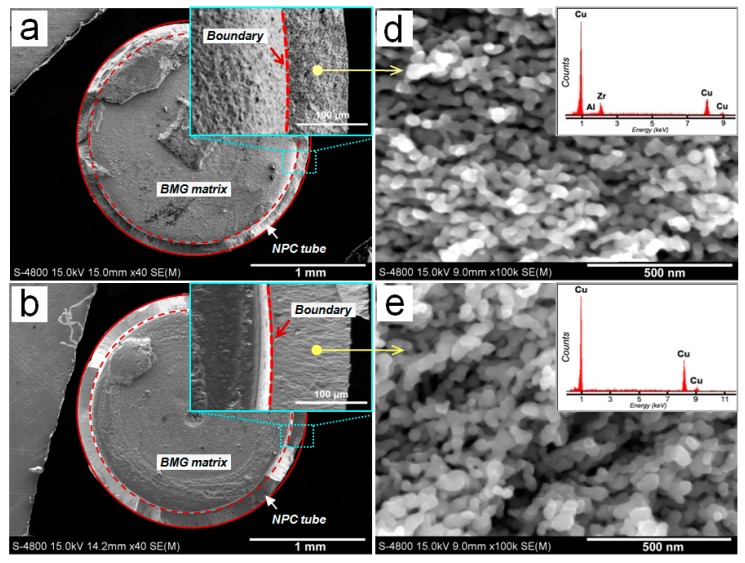

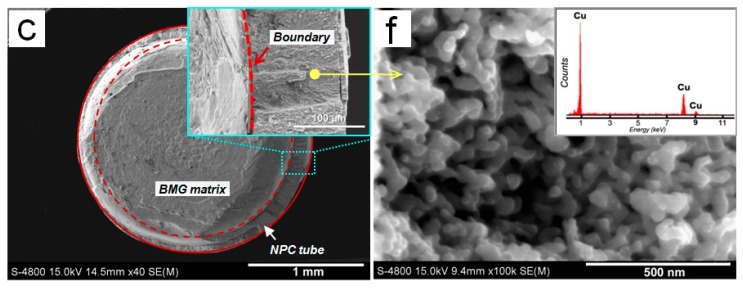
(**a**–**c**) The section views of the nanoporous copper (NPC)/BMG composite rod 1, 2, and 3, respectively; (**d**–**f**) High magnification SEM images with their corresponding EDS spectra taken from the same place (about 60 μm away from the NPC surface).

**Figure 4:**
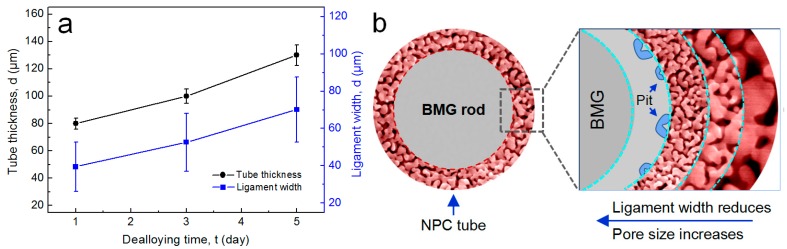
(**a**) Changes in the NPC tube thickness and NPC ligament width with dealloying time; (**b**) Schematic illustration of the hierarchical architectures along the dealloying depth.

The cross-sectional SEM images of the NPC/BMG composite rods are shown in Figure 3a–c. Obviously, it can be seen that the inner BMG matrix core is homogeneously coated by the outer tube-shaped NPC layer with a certain thickness. Moreover, the cross-sectional morphologies exhibit that the NPC tubular layer smoothly joints to the BMG matrix. From the inserts of Figure 3a–c, it is found that the thickness of NPC tube increases with increasing leaching time in HF solutions. As plotted in Figure 4a, the average thickness of the NPC tube for the NPC/BMG composite rod 1, 2 and 3 is 85, 110 and 135 μm, respectively. Figure 3d–f represents high magnification SEM images with their corresponding EDS spectra taken from the same place (where is about 60 μm away from the NPC surface). Apparently, three-dimensional (3D) ligament-channel structures interpenetrate throughout the whole NPC tube for all the composites. Although all metallic ligaments are mostly similar in morphology, the distinct ligament coarsening with the prolongation of dealloying time can be readily observed. The mean ligament width measured at the same place where we select (Figure 3 and Figure 4a) gradually increase from ~45 nm for NPC/BMG rod 1 to ~70 nm for NPC/BMG rod-3. Similar results were reported for nanoporous copper produced by dealloying of amorphous Cu-Hf-Al alloys in 0.5 M HF for different time [30]. On the other hand, from the sectional SEM images of NPC/BMG rod 1, 2, and 3, it is observed that the NPC composites demonstrate a hierarchical architecture with ligament width and nanopore size gradient through the whole NPC tubular depth. The nanopore size gradually increases from the outer layer to the inner layer of the NPC tube, whereas the ligament width decreases. Figure 4b illustrates the schematic section views combined with the hierarchical architectures along the dealloying depth for the NPC/BMG composite rods. The formation mechanism of the hierarchical nanoporous structure by dealloying Cu-Zr-Al BMG rod was systematically studied in our previous work [25]. The NPC/BMG composite rods with tailored NPC tube thickness and hierarchical nanoporous copper covering on the BMG core have been facilely fabricated by modulating the dealloying time of the Cu_50_Zr_45_Al_5_ BMG rod in 0.05 M HF solution.

### 3.2. Mechanical Properties of NPC/BMG Composites

**Figure 5:**
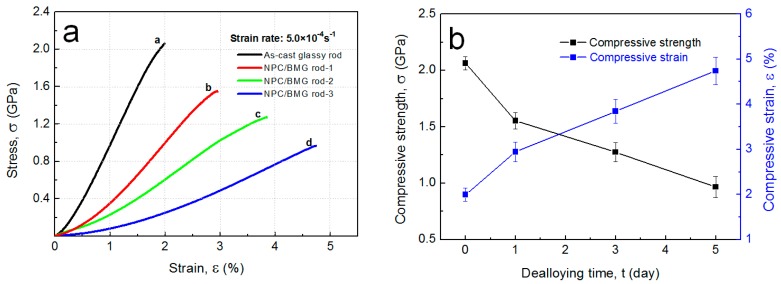
(**a**) Compressive stress-strain curves of the as-cast Cu_50_Zr_45_Al_5_ BMG rod and the NPC/BMG composite rods; (**b**) Changes in the compressive strength and compressive strain of NPC/BMG composite rods with dealloying time.

Figure 5a shows the stress-strain curves under a compressive applied load for the Cu_50_Zr_45_Al_5_ BMG rod and the NPC/BMG composite rods of 2 mm in diameter. For the Cu_50_Zr_45_Al_5_ BMG, the compressive fracture strength (*σ*_f_) and compressive fracture strain (*ε*_f_) are 2.1 GPa and 2.0%, respectively, which is consistent with those data for the Cu-based BMGs [31,32]. Although a decrease in the compressive strength is observed for the NPC/BMG composite rods, the NPC composites still retain high mechanical strength values of 0.9~1.5 GPa, as compared to the alloy foams [33,34,35,36], BMG foams [8,9], and NPG [37,38] composites. Moreover, the NPC/BMG composite rod 1, 2 and 3 exhibit large compressive strains of ~2.9%, ~3.8%, and ~4.7%, respectively, which is superior to that for the Cu_50_Zr_45_Al_5_ glassy rods. It is believed that the excess free energy generated within a shear band during deformation under an applied load is highly unstable and easily facilitates the development of shear cracks. In this regard, the outer tube-shaped NPC layer with a certain thickness can act as a buffer function zone to absorb the excess free energy, which limits shear band extension and avoids cracks progression of the inner Cu-Zr-Al BMG matrix [25]. Meanwhile, from Figure 5b, it is seen that the compressive strain of the NPC/BMG composite increases with an increase in the dealloying time. This result suggests that the thicker nanoporous copper tubular layer resulting from the extension of dealloying time may possess a stronger buffer capacity to delay the formation and propagation of shear cracks.

Obviously, the mechanical behavior of the presented NPC/BMG composites is different from those of the BMG foams [8,9] or the other BMG composites [5,6,7]. The NPC/BMG composite rods possess a unique architecture of the inner rod-shaped BMG core enfolded with the outer tube-shaped NPC layer. The material design of the new-type 3D nanopores/BMG composites is totally different with the previous reinforced second phases BMG composites [5,6,7] or pores-containing BMG foams [8,9]. In macroscopical view, the BMG substrate with highly uniform dispersion of pores or second phases cannot be simply regarded as the pure BMG any more, whereas the NPC/BMG composites possess the distinct phase separation between the inner glassy metal core and the outer NPC layer. Moreover, the NPC composite rod is different from the submicron size thin film [10,12]. The size effect for the thin film strongly influences the mechanical behavior. Thereby, the present NPC composite rods do not follow the rules suited for the traditional composite materials or thin films. The mechanical behavior exhibits different behavior as well. Since the outer NPC layer is a soft and ductile phase, it is reasonable to infer that the fracture strength of the NPC composite rods is still dominated by the metallic glass core that provides the much higher strength as compare to the NPC foams. On the other hand, the outer NPC layer just absorbs more excess free energy within the shear band generated from the inner metallic glass core, thus alleviates the formation and progression of shear cracks. Furthermore, we measured the compression test many times, and the stress-strain curves have a very good reproducibility and a similar trend, which proves the present data are reliable. For elastic behavior, it may be interpreted that the elastic behavior of the NPC/BMG composite rods are aptly named as a “pseudo-elasticity behavior.” The more detailed work will be challenged in future.

**Figure 6:**
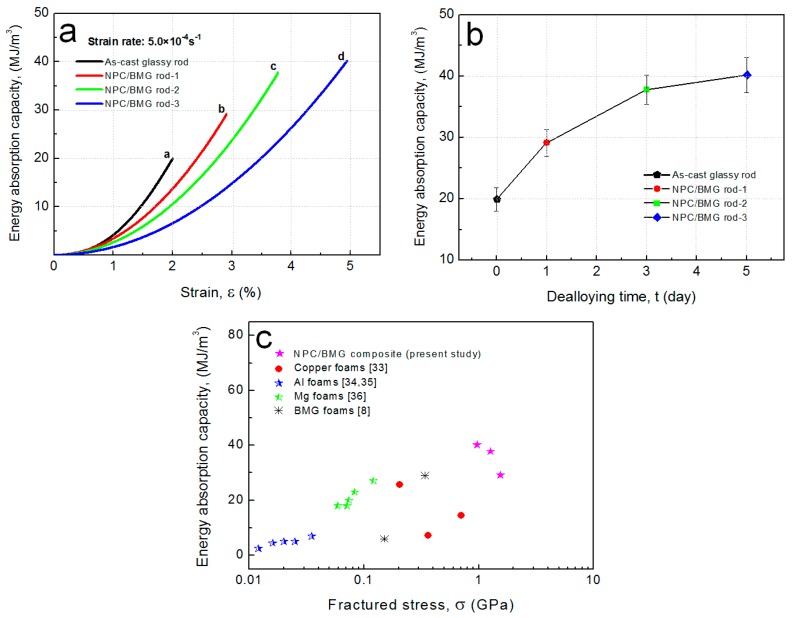
(**a**) Energy absorption capacity of the as-cast Cu_50_Zr_45_Al_5_ BMG rod and the NPC/BMG composite rods; (**b**) Changes in the energy absorption capacity of composite rods with dealloying time; (**c**) The comparison of energy absorption capacity (EAC) for NPC/BMG composite rods and other alloy foams.

Based on the stress-strain curves, we can further examine the energy absorption capacity (EAC), which is calculated by the area under a stress-strain curve according to the Equation (1) [39], as follows:

(1) $$W=\int_{0}^{\varepsilon }\sigma d\varepsilon$$

where *W* is the energy absorption capacity, *σ* is the stress and the stain is *ε*. As shown in Figure 6a,b, the EAC values of the NPC/BMG rod 1, 2, and 3 are 29.1, 37.8, and 40.2 MJ/m^3^, respectively, while the Cu_50_Zr_45_Al_5_ glassy rod exhibits the maximal EAC of 19.9 MJ/m^3^. The results indicate that the NPC/BMG composite rods possess much higher energy absorption capacity than the Cu_50_Zr_45_Al_5_ glassy rods due to the existence of the NPC tubular layer. Furthermore, Figure 6c summarizes the energy-absorption capacity of the present NPC/BMG composites, copper foams [33], Al foams [34,35], Mg foams [36], and BMG foams [8]. It appears that the present NPC/BMG composite rods show good energy absorption performance as compared with the existing porous solids including metal foams and BMG foams. The origin of the high-energy absorption performance can be interpreted by three factors: (1) the relatively high strength of the parent BMG materials, (2) the exterior NPC tubular layer with a uniform nanoporous structure acting as a plastic shielding for reducing the formation of the shear cracks [25], and (3) the beneficial architecture design for the NPC/BMG composites combined with inner amorphous phase core and outer NPC tubular layer. Accordingly, the NPC/BMG composite rods with excellent mechanical properties as well as good energy absorption performance are a promising candidate in energy absorption fields.

### 3.3. The Correlation of Microstructure and Mechanical Properties

**Figure 7:**
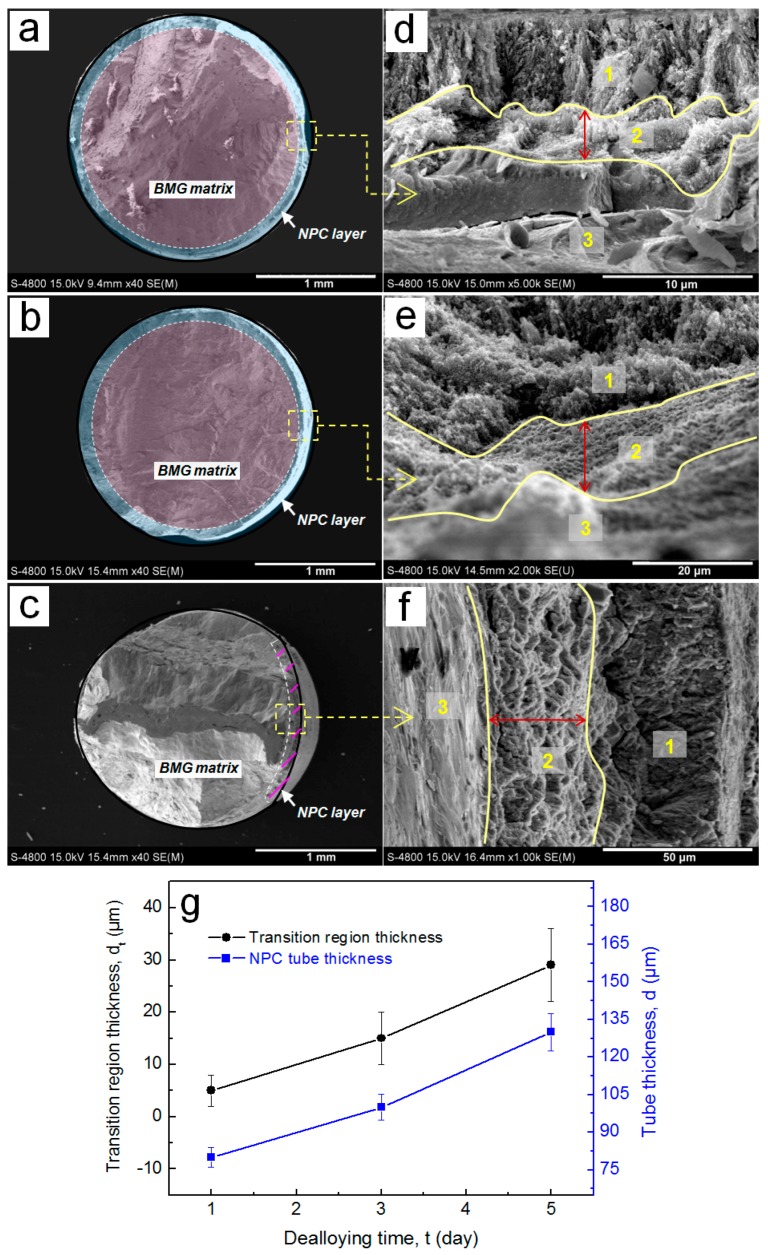
SEM images showing the fracture morphologies of the NPC/BMG composite rods subjected to the compression testing: (**a**–**c**) The cross-sectional morphology of the NPC/BMG rod 1, rod 2, and rod 3, respectively; (**d**–**f**) Corresponding high magnification images of a–c (the arrows show the observation position), all fractured composite rods exhibiting three distinct characteristics from the glassy matrix to NPC; (**g**) Changes in the NPC tube thickness and transition region width with dealloying time.

To clarify the correlation of nanoporous structure and mechanical properties, the deformation morphologies on the cross section of the NPC/BMG composite rods after compression testing are shown in Figure 7. In Figure 7a–c, it is clearly observed that the fractured tubular layer with different thickness covers on the glassy alloy core. Although the NPC encounters a certain deformation under a load, the NPC/BMG composite rods still maintain a tight junction between the BMG matrix and NPC. On the other hand, Figure 7d–f exhibit that the fracture surface of the composites can be classified into three distinct characteristics: (1) the outer NPC (zone 1), (2) the transition region between BMG and NPC (zone 2), and (3) the inner BMG (zone 3). Meanwhile, it is worth noting that the width of the transition region (marked by red double arrow in Figure 7d–f) reveals a similar increasing trend with the thickness of the outer NPC tubular layer. Figure 7g shows the change in the thickness of NPC tube and the width of the transition region with dealloying time. As the thickness of NPC tubular layer increasing, the interactions between the outer NPC tube and the inner BMG matrix is likely to be enhanced for the NPC/BMG composite rod, which results in the formation of the wider transition zone. Therefore, it can be concluded that the extension of dealloying time results in the formation of the thicker NPC layer and wider transition zone, which is attributed to the larger compressive strain.

**Figure 8:**
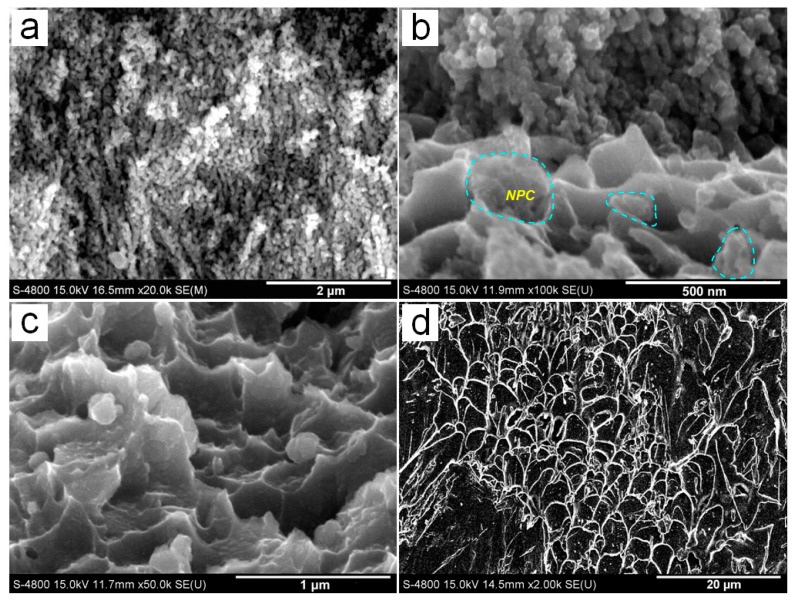
The enlarged fracture deformation structure of the NPC/BMG composite rod-3 in Figure 7e: (**a**) The deformed NPC ligament from zone 1; (**b**) The nano-sized dimples with NPC fragments (marked by circles) from zone 2; (**c**,**d**) The deformation structure of the glassy matrix from zone 3.

For further understanding the buffer function of the NPC tubular layer under loading, the enlarged fracture deformation structure of the NPC/BMG composite rod 3 is observed in Figure 8. In the high-magnification image of NPC zone (Figure 8a), it is clearly seen that the random distributional NPC ligaments turn to be oriented along the shear stress plane, reflecting a large deformation occurs for the outer NPC tubular layer. Furthermore, the middle transition zone (Figure 8b) that connects both the NPC and the glassy matrix consists of the mixture of many nano-sized dimples with some NPC fragments. It should be noted that the dimple-like deformed structure appears in the inner BMG matrix adjacent to the transition zone (Figure 8c). By contrast, the fracture surface of the inner glassy matrix shown in Figure 8d is characterized by a homogeneous distribution of vein-like patterns that the typical monolithic BMGs exhibit. No dimple patterns are observed on the inner glassy matrix. Based on both the deforming appearance of nano-sized dimples in Figure 8b and dimple-like patterns in Figure 8c, it gives clear evidence that the outer NPC tubular layer undergoes a strong interaction with the inner BMG matrix rod under an applied load. On the other hand, it was reported that the size of dimples on the fracture surface can be used as an important parameter to evaluate a large strain of metallic materials [40,41]. As compared with the NPC/BMG composite rod 1, the NPC/BMG composite rod 3 samples exhibit an enlarged dimple-like structure (Figure 8b,c), implying an improved compressive strain of NPC/BMG composite rods.

Generally speaking, the bulk metallic glasses (BMGs) easily fail catastrophically due to the rapid propagation of the highly localized shear bands. The excess free energy within the shear band can be associated with a free volume chemical potential that provides a driving force for free volume coalescence, void nucleation and formation of shear crack during shear deformation of metallic glasses [42,43]. In order to solve this issue, the new-type NPC/BMG composites with good cooperation of the inner rod-shaped Cu-based BMG matrix and the outer NPC tubular layer is well designed. Usually, the interface between the metal substrate and coating film will generate an intensely stress localization [44]. However, the present NPC composite is totally different. The outer dealloyed layer exiting nanoporous structure is soft and ductile phase. During compression testing, there is much smaller stress yielded in the interface between the glassy core and dealloyed layer. Moreover, it should emphasize that the outer NPC layer possesses an inherent feature of the outstanding energy absorption ability. From Figure 6a,b, it can be seen that the energy absorption ability of the NPC composites increases with an increase in the NPC thickness. When the NPC/BMG composite rod is subjected to the compression and the shear propagation, it is believed that the wider nanoporous layer (as indicated in Figure 8b,c) can interact more strongly with the inner BMG matrix to absorb more excess free energy within the shear band, thus alleviating the formation and progression of shear cracks arisen from the inner Cu-Zr-Al glassy matrix. Furthermore, the NPC layer also can act as a buffering shielding for reducing the residual stress or the stress concentration at the shear crack tip of glassy matrix by absorbing the high local energy. For instance, the present composite rod is similar to a metal rod wrapped with polyfoam.

On the other hand, from SEM images in Figure 3a–c, Figure 7 and Figure 8, it was found that the changes in the width of transition zone after fracture are dependence on the thickness of the dealloyed zone. By contrast, the deformed NPC metals obtained for different dealloying time after compression exhibit the similar morphologies [25]. Therefore, it can be concluded that the fracture behavior of the composites is affected by the thickness of outer dealloyed zone, while the morphology and the pores connection (Figure 2) act as the minor factors. Thereby, the thicker tube-shaped NPC layer as a buffer zone can perform a better buffer function to delay the occurrence and development of a shear crack, which further relieves the sudden fracture of the NPC/BMG composites.

**Figure 9:**
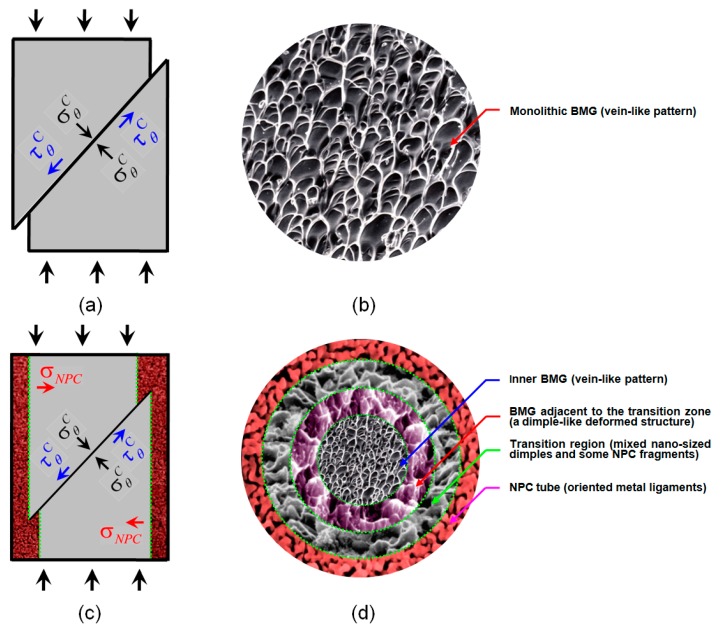
Illustration of the fracture deformation processes for (**a**,**b**) monolithic BMG and (**c**,**d**) NPC/BMG composite rods under applied load.

According to the discussions above, we schematically illustrate the compressive fracture processes of monolithic BMG and the NPC/BMG composite in Figure 9. Under compressive loading, the normal stress $\sigma_{\theta }^{C}$ always exerts on the fracture plane in a compressive mode [45]. Consequently, the fracture process of monolithic BMG or BMG matrix composites should be mainly controlled by the shear stress $\tau_{\theta }^{C}$, as shown in Figure 9a,c. Usually, the compressive fracture surfaces of a monolithic BMG exhibit a vein-like structure feature with a rather uniform arrangement (Figure 9b). This vein-like structure is attributed to local softening or melting within the shear band induced by the high elastic energy upon instantaneous fracture [46,47]. The soft or molten metallic glass within the shear bands easily flows and appears in a vein-like structure feature. By contrast, in addition to the shear stress $\tau_{\theta }^{C}$ and normal stress $\sigma_{\theta }^{C}$ exerted on the fracture plane of the NPC/BMG rod, an extra inward force $\sigma_{NPC}$ on inner BMG matrix (Figure 9c) can be generated from the buffering shielding of outer NPC tube. Figure 9d qualitatively illustrates the four distinct deformation characteristics of the fracture surface for the NPC/BMG composite rods: (1) the outer NPC tube with oriented metal ligaments, (2) the transition region with mixed nano-sized dimples and some NPC fragments, (3) the glassy matrix adjacent to the transition zone with the dimple-like deformed structure, and (4) the inner glassy matrix with a uniform vein-like pattern. Since the present composite rods are new architecture materials (inner rod-shaped amorphous phase core and outer tube-shaped NPC layer), it is very complicate to give quantitative explanations about the fracture deformation mechanics, which leaves a big challenge topic for future studies.

## 4. Conclusions

In summary, we report a systematic study of the structural hierarchy, mechanical behaviors, and energy absorption capacity (EAC) of the NPC/BMG composite rods with different immersion time in HF solutions. Prolonging the dealloying time is found to be an effective way to increase the thickness of NPC tube, which subsequently enhances the compressive strain and energy absorption capacity of the NPC/BMG composite rods. SEM observation shows that the fractured surface of composite rods display four distinct deformation characteristics, especially with a unique transition zone. As the thickness of NPC tubular layer increasing, the interactions between the outer NPC tube and the inner BMG matrix is likely to be enhanced, resulting in the formation of the wider transition zone. Meanwhile, the thicker NPC tube can play a better buffer function for alleviating the formation and development of shear cracks arisen from the inner glassy matrix, and further delaying the fracture of the NPC/BMG composites. The new developed 3D nanoporous BMG rods with unique architecture and useful properties show highly promising prospect in multifunctional applications.
